# Diabetes self-management in three different income settings: Cross-learning of barriers and opportunities

**DOI:** 10.1371/journal.pone.0213530

**Published:** 2019-03-19

**Authors:** Jeroen De Man, Juliet Aweko, Meena Daivadanam, Helle Mölsted Alvesson, Peter Delobelle, Roy William Mayega, Claes-Göran Östenson, Barbara Kirunda, Francis Xavier Kasujja, David Guwattude, Thandi Puoane, David Sanders, Stefan Peterson, Göran Tomson, Carl Johan Sundberg, Pilvikki Absetz, Josefien Van Olmen

**Affiliations:** 1 Department of Public Health, Institute of Tropical Medicine, Antwerp, Belgium; 2 Department of Primary and Interdisciplinary Care, University of Antwerp, Antwerp, Belgium; 3 Department of Public Health Sciences, Karolinska Institutet, Stockholm, Sweden; 4 Department of Food Studies, Nutrition and Dietetics, Uppsala University, Uppsala, Sweden; 5 School of Public Health, University of the Western Cape, Belville, South Africa; 6 Chronic Disease Initiative for Africa, University of Cape Town, Cape Town, South Africa; 7 Department of Epidemiology and Biostatistics, School of Public Health, College of Health Sciences, Makerere University, Kampala, Uganda; 8 Department of Molecular Medicine & Surgery, Diabetes and Endocrine Unit, Karolinska Institutet, Stockholm, Sweden; 9 Department of Learning, Informatics, Management & Ethics, Karolinska Institutet, Stockholm, Sweden; 10 Department of Physiology & Pharmacology, Karolinska Institutet, Stockholm, Sweden; 11 Collaborative Care Systems Finland, Helsinki, Finland; Institute of Public Health and Clinical Nutrition, University of Eastern Finland, Kuopio, Finland; Florida International University Herbert Wertheim College of Medicine, UNITED STATES

## Abstract

The burden of type 2 diabetes is increasing rapidly, not least in Sub-Saharan Africa, and disadvantaged populations are disproportionally affected. Self-management is a key strategy for people at risk of or with type 2 diabetes, but implementation is a challenge. The objective of this study is to assess the determinants of self-management from an implementation perspective in three settings: two rural districts in Uganda, an urban township in South Africa, and socio-economically disadvantaged suburbs in Sweden. Data collection followed an exploratory multiple-case study design, integrating data from interviews, focus group discussions, and observations. Data collection and analysis were guided by a contextualized version of a transdisciplinary framework for self-management. Findings indicate that people at risk of or with type 2 diabetes are aware of major self-management strategies, but fail to integrate these into their daily lives. Depending on the setting, opportunities to facilitate implementation of self-management include: improving patient-provider interaction, improving health service delivery, and encouraging community initiatives supporting self-management. Modification of the physical environment (e.g. accessibility to healthy food) and the socio-cultural environment (i.e. norms, values, attitudes, and social support) may have an important influence on people’s lifestyle. Regarding the study methodology, we learned that this innovative approach can lead to a comprehensive analysis of self-management determinants across different settings. An important barrier was the difficult contextualization of concepts like perceived autonomy and self-efficacy. Intervention studies are needed to confirm whether the pathways suggested by this study are valid and to test the proposed opportunities for change.

## Introduction

Non-communicable diseases (NCD) are strong contributors to poverty and inequity within and across countries, disproportionately affecting people of low socioeconomic status [1]. A recent series of articles in the Lancet launched a strong call for action against the burden of NCDs [2], directly in line with Sustainable Development Goal (SDG) 3·4 to reduce premature NCD mortality and indirectly in line with SDGs 1, 2, 4, 5, and 10 [1]. Type 2 diabetes (T2D) is a major contributor to the NCD burden. Similar to other NCDs, the global prevalence of diabetes in adults is increasing and is estimated to grow from 8·8% in 2015 to 10·4% in 2040 [3], with Sub-Saharan Africa contributing the largest share of this growth [3]. In high income countries (HICs), socio-economically disadvantaged populations and immigrants are disproportionately affected [4].

Self-management is one of the key elements for adequate prevention and treatment of T2D and other NCDs [5]. It improves care processes and health outcomes, for instance through improved treatment adherence and adaptation of treatment to a person’s situation [5,6]. Self-management means that individuals play an active role in managing their condition. This implies that they engage in decision-making, adopting and adapting strategies to improve their health status regarding that particular condition [7]. It also suggests an engagement in supportive partnerships with other people, such as family, friends, health providers, community members, and peers [7]. To realize the latter, individuals need to adopt a pro-active mind-set, skills, and knowledge. Beyond the individuals’ engagement, this requires the “right” conditions with regards to the health system, the socio-cultural and physical environment, and their family and friends, also categorized as self-management support [8].

Adopting self-management remains a challenge for people living with T2D in both HICs [9] and low and middle income countries (LMICs)[10]. One of the reasons is that it requires an approach tailored to a particular population and context [11]. This requires information on the context-specific determinants and status of self-management, and on the components of self-management support.

The determinants of self-management are usually assessed within the comprehensive package of care for chronic diseases using the chronic care model or a modified version [12]. These models do not adequately include the individual behavioral mechanisms that play an essential role in self-management. Behavior change models, on the other hand, focus on the individual pathways of behavior, but do not include the specific actors and health system elements. In this study, we use a framework that connects–from a perspective of chronic conditions–essential mechanisms of behavior change, a comprehensive analysis of relevant actors, the proximal environment including the community, and the health care environment.

This study aims at assessing determinants of self-management using the proposed framework in three different settings–rural Uganda, an urban township in South Africa, and socioeconomically disadvantaged suburbs with a predominant immigrant population in Sweden. Furthermore, this study aims at identifying opportunities to improve self-management through learning from these different contexts.

The selected settings offer a potential for reciprocal learning because of their contextual characteristics, such as: income level, role of the community, quality of health care, and experience with other chronic diseases (e.g. HIV/TB in South Africa & Uganda) [13]. Examples of questions for cross-lessons based upon those contextual specifics are: which successful complementing self-management support activities emerge from an under-resourced health system setting (lessons from Uganda)? How can community-based initiatives strengthen self-management (lessons from South Africa and Uganda)? How can facility-based care for prevention and control contribute to self-management of vulnerable groups (lessons from Sweden)?

The study is part of the formative phase of the SMART2D project: “A person-centred approach to Self-Management And Reciprocal learning for the prevention and management of Type 2 Diabetes”. The SMART2D project was funded by the European Union (Horizon 2020), and aims to improve self-management for people at risk of or living with T2D [14]. The development and application of the framework has informed the selection and implementation of self-management strategies in each study site.

## Methods

The SMART2D study aims for cross-contextual reciprocal learning in three cycles [13]. The studies in this paper describe the first learning cycle which had three steps. The first step was to build a conceptual framework that fosters a common understanding in the three settings throughout the SMART2D project. In a second step, this common framework was translated concurrently into a generic topic guide and site-specific focus group and interview guides (S1 and S2 Appendix). Site-specific data collection (focus groups, interviews, and observations) was carried out by each of the country teams and preliminary data-analysis was conducted. In a third step, each of the sites populated the themes of the generic topic guide that were applicable to their specific site, based on the data collected in the previous step and additional secondary data (i.e. national statistics, findings from other studies, and project documents). This data was synthesized in a table with cross-cutting themes and a core team assessed commonalities and differences in self-management and its influencing factors which forms the subject of this paper.

### Development of a transdisciplinary framework and a topic guide

A common framework was developed to guide site-specific data collection and to develop a generic topic guide. The development of this transdisciplinary self-management framework (hereafter referred to as the “SMART2D framework”) followed an iterative process with inputs from the literature and from researchers from different disciplines during consortium meetings and workshops.

The first step in the development of this framework was a critical review of the literature [15]. We sought to identify the most significant elements (including systems, actors, the environment, the individual) that determine self-management in people living with T2D. In particular, we were looking for studies presenting novel theories and conceptual frameworks. Only theories that were based on empirical evidence were considered, although, no formal quality assessment was done. Studies were identified through the use of search engines like Google scholar and Pubmed, using search terms identified through brainstorming sessions with the research team. Search terms included keywords like: “self-management”, “health systems”, “chronic conditions”, “non-communicable diseases”, “models”, “frameworks”, etc. Search terms were iteratively added and refined with input from collaborating researchers and the identified literature (pearl-harvesting). The search process also included: browsing, “consulting peer experts,” “Snowball” methods such as pursuing references of references, and electronic citation tracking which are known to be powerful for identifying high quality sources in obscure locations [16]. For complex and heterogeneous evidence (such as those undertaken for management and policymaking questions) formal protocol-driven search strategies may fail to identify important evidence, while informal approaches such as the ones used in this search process can substantially increase the yield and efficiency of search efforts [16]. Search results were sorted by relevance and studies were selected based on their potential conceptual contribution.

From the selected studies, we extracted elements or theories that determine self-management and are relevant for T2D. In particular, we focused on mechanisms that explain the individuals’ behavior but are related to their environment or health system. The retrieved elements and theories were discussed in a core research team consisting of the following researchers: a behavioral expert (PA), health systems experts (JVO, JDM, JA, MD), an endocrinologist (CGÖ), and researchers with site-specific expertise from Uganda (RWM), South Africa (PD) and Sweden (HMA). Selected theories and elements were brought together in an initial framework describing the determinants and mechanisms of self-management, which was then presented to the SMART2D consortium. Discussions led to modifications and the present framework is the end result of this process. Theories were selected based on their relevance to self-management among people with chronic conditions (from a multidisciplinary perspective), and relevance to the implementation of self-management. Through the combination of perspectives from different disciplines, this framework brings about a new way of looking at how self-management works beyond the traditional perspective of each of those disciplines. For example: health systems thinking, is connected to individual behavior through individual behavioral mediators.

The initial framework was presented to the country research teams of the consortium during a workshop (that all together comprised 21 members) to discuss the relevance and usability of the framework in each of the study contexts. The discussions involved brainstorming on the role of context-specific factors (i.e. actors, community structures, platforms, partners and strategies associated with self-management). Further development and refinement of the framework continued through a series of workshops and conference calls facilitated by JDM and JVO, held separately for each of the three country research teams until a final version was approved.

The framework integrates behavioral change theories with mediation through latent variables [17], chronic care models [18,19], health systems theory [20], and the influence of the proximal environment to a common perspective that “transcends” the initial perspective of each of the specific disciplines.

The framework is based on the idea that self-management behavior results from a continuous and reciprocal interaction between the individual and the individual’s proximal environment which includes the health system, a socio-cultural component and a physical component.

**Fig 1:**
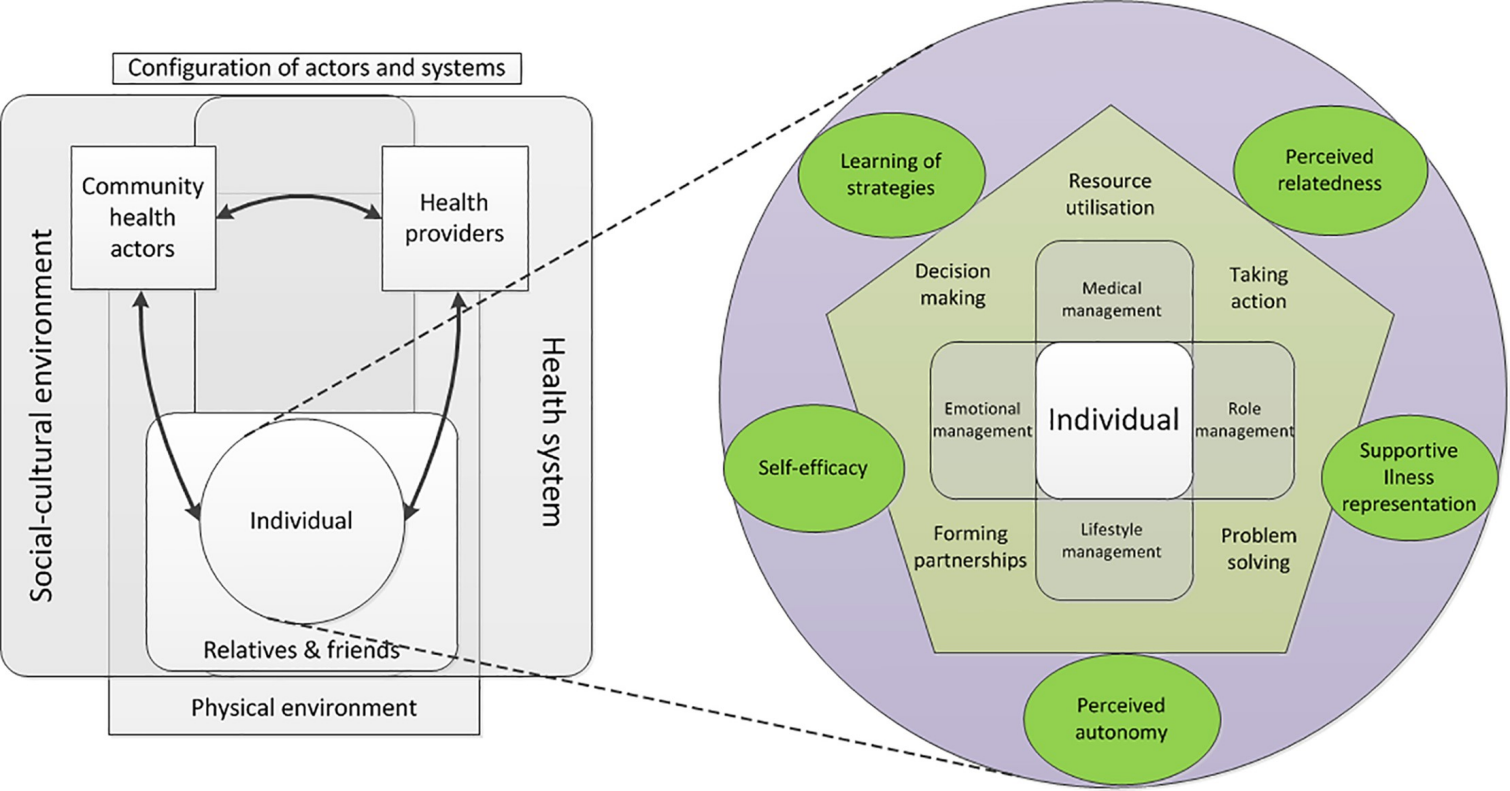
The SMART2D self-management framework presenting the different elements that determine self-management. Legend: Zooming in on the individual reveals mediating factors (in green oval shapes), self-management skills (in the pentagon), and self-management tasks (at the core).

As such, the framework integrates actors and systems that are considered to play a determining role in self-management (Fig 1; left side; “configuration of actors and systems”). The individual at risk of, or living with T2D has a central role in this configuration of actors and systems and is closely connected to their family and friends. As presented by the innovative care for chronic conditions framework, the individual belongs to an actors’ triad with community health actors and health providers [19]. Each of those actors interact with the health system, the physical environment, and the socio-cultural environment.

When focusing on the individual (Fig 1; right side) the framework distinguishes three groups of individual or intrapersonal factors: mediating factors at the outer circle, self-management skills in the pentagon, and self-management tasks at the core. The reason to distinguish among these factors is that they have a different function in the implementation of self-management.

The four core self-management tasks (medical management, emotional management, role management, and lifestyle management) positioned at the core of the framework represent self-management behavior and were adopted from Corbin and Strauss [21]. Corbin and Strauss identified three sets of tasks through a qualitative study on the work of people with chronic conditions. What we call lifestyle management in our framework is part of medical management in their classification. Adequate execution of these core self-management tasks results in self-management behavior, requires the five self-management skills, and is facilitated by the five mediators. From an implementation perspective, these tasks should be kept in mind as an end goal, but improvement of these tasks ideally takes place through interventions that address the individual mediators. The five self-management skills were introduced by Lorig and Holman (decision-making, resource utilization, taking action, problem solving, and forming partnerships)[7]. Adoption of these skills is required for the adequate execution of the specific self-management tasks, depends primarily on the initiative of the individual, and is facilitated by the five mediators. Therefore, from an implementation perspective, the adoption of the skills ideally happens through addressing these mediators. Finally, these mediators link the individual’s self-management skills and tasks with their interactions with their proximal environment (Fig 1; “configuration of actors and systems”), which implies that these mediators strongly depend on the environment. Appropriate implementation of self-management should therefore create an environment that fosters change through addressing these mediators when targeting self-management skills or tasks. The five mediators include perceived autonomy, perceived relatedness, and self-efficacy (Box 1), which are identified by Ryan and Deci’s self-determination theory as the three basic psychological needs that foster high quality forms of motivation and engagement, and hence play an important role in the adoption of healthy behavior [17]. Illness representation as defined by Leventhal corresponds to the individual’s understanding of T2D through personal experience, socio-cultural information, and healthcare interactions [22,23]. Learning of strategies refers to acquiring knowledge and understanding of self-management strategies and skills through thought, own experience, and perception (Box 1).

**Box 1. Definitions of the individual mediators of self-management.**

**Perceived autonomy** corresponds to the individual regulating his/her behavior with the experience of choice and reflective self-endorsement, while experiencing external pressure to act in a certain way would make her/him feel less autonomous [17].

**Perceived relatedness** corresponds to the need of feeling connected to and cared about by others [17].

**Self-efficacy** was initially defined by Bandura as “people's beliefs about their capabilities to produce designated levels of performance that exercise influence over events that affect their lives”[24]. Self-determination theory uses the term perceived competence, but the concept corresponds to Bandura’s self-efficacy [17].

**Illness representation** can trigger actions to reduce health risk and thus change the individual’s behavior, based on the model developed by Leventhal [22]. This model proposes five core elements: (1) identity refers to the individual’s awareness of signs and symptoms of the disease; (2) cause refers to the individual’s idea of the cause of the condition; (3) timeline refers to how long the condition might last according to the individual; (4) consequences refers to the individual’s ideas about the potential consequences of the condition on her/his life; and (5) control corresponds to whether the condition can be cured or kept under control and the degree to which the individual can take part in this [22].

**Learning of self-management strategies** includes both the acquiring of knowledge and the development of skills. The learning process corresponds to active learning which occurs when a person takes control of his/her own learning experience. This active learning process can happen through cognitivism (internal processing of information), or constructivism (new information is linked to prior knowledge, leading to a subjective mental construct). In particular, we want to stress the value of social constructivism in self-management: learning takes place because of the interaction with others (e.g. peers, community members, relatives, etc.)[25].

### Translation of the SMART2D framework to a topic guide

The constructs presented in the framework were translated into a generic topic guide (S2 Appendix). This translation process was done by a cross-site coordination team comprising of a behavioral scientist (PA, facilitating intervention development) and three health systems researchers (JVO & JDM facilitating cross-country lessons and MD facilitating conceptualization and implementation); and country teams lead by RWM, PD & HMA in Uganda, South Africa, and Sweden respectively. The topic guide covered information related to self-management support to be sourced from site-specific primary data collected through focus group discussions, individual interviews, observations, and other relevant secondary data.

Regarding the individual, the guide focuses on the characteristics of the studied populations and individual mediators. Regarding family and friends, the guide explores how they support the individual. Regarding the health providers, the focus is on interpersonal quality of care of public primary care providers. Regarding the community health actors, the focus is on identifying relevant community initiatives and their link with health providers. Regarding the health system, the focus is on aspects of service delivery (i.e. accessibility, quality of care, continuity of care, type of care). Regarding the socio-cultural and physical environment, the focus is on elements that influence physical activity and healthy diets.

### Data collection

Concurrent data collection using the site-specific focus group and interview guides (S1 Appendix) and the generic topic guide (S2 Appendix) was informed by the SMART2D framework presented in the first paragraph of this section. In-depth interviews, FGDs and observations were conducted in each site from March to August 2015 and preliminary data analysis was done side-by-side to inform the topic guide. Table 1 provides a summary of participants’ details, recruitment, and data collection of the primary data in each site (also published or submitted elsewhere as indicated in the table). Concurrently, from March to December 2015, data were collected using the generic topic guide and following an exploratory and multiple case study design, which allows exploring self-management within its real-life context through the concurrent use of different sources of information and data collection methods [26]. Data pertaining to three cases were collected: 1) an urban township in Cape Town, South Africa; 2) socioeconomically disadvantaged suburbs in Stockholm County, Sweden; 3) a rural area comprising of Iganga and Mayuge district, Uganda. All processes described henceforth refer to data collected through the generic topic guide.

**Table 1:** Site-specific participant recruitment and data collection methods.

Country	Participants	Number of participants		Recruitment principle	Data collection procedures	Main themes in the site-specific interview/FGD guide
**Sweden**		**Male**	**Female**			
	People diagnosed with T2D	6	6	- People diagnosed with T2D or at risk of T2D were identified from a patient database from the participating health centers and each participant was contacted by a diabetes nurse. Interested participants were scheduled for interviews by the research team. In addition, people at risk were identified through a T2D screening program run by the 4D project and jointly managed by Karolinska Institute and the Stockholm County Council[25]. Selection of participants was based on the following criteria: (a) men and women aged 30–75 years and born outside Europe, (b) at risk of /diagnosed with T2D (c) lived with T2D or risk for six months and more (d) lived in Sweden for at least five years and (e) living in socio-economically disadvantaged communities.	- 12 individual interviews were conducted among participants diagnosed with T2D and 18 among participants at risk of T2D. Participants varied in gender, age, (30–75) and country of birth (from the Middle East, South-America, and Africa. Informed consent was sought from participants prior to the interview. Interviews were conducted in Swedish by two research team members lasting 45–90 minutes. All the participants received two movie tickets after participation. Based on Malterud et al’s description of information power [27], the study sample provided sufficient information power to address the research questions. Further details on recruitment and data collection process of participants with T2D and health providers are published elsewhere [28].	- For people diagnosed with T2D, four major themes were explored: Perceptions of diabetes diagnosis, patient and provider interactions, experiences of diabetes self-management, and support for self-management.
	People at risk of T2D	10	8			- For people at risk of T2D, the following themes were explored:—Perceptions of risk for diabetes, Care for persons at risk of diabetes and interaction with healthcare providers, experiences of coping with being at risk of diabetes and support for persons at risk of diabetes.
	Healthcare providers (including: doctors, nurses & healthcare managers)	2	9	- Health providers were purposively sampled to include doctors and nurses who had frequent contact with T2D patients.	- 3 group interviews were conducted with 3 doctors and 5 diabetes nurses who had daily or weekly contact with diabetes patients, 1 group interview was held with 4 health managers of the participating health centers. The participants signed an informed consent form prior the interview. The interviews were conducted in Swedish by two members of the research team. One moderated the discussions and the other took notes and recorded the session. The interviews lasted between 45–60 minute. Participants received two movie tickets for participation. The study sample provided sufficient information power to address the research questions based on Malterud et al’s description of information power [27].	- Main themes covered included: structure and process of diabetes care at the health center, patient and provider interactions and caregivers’ experiences in managing T2D patients and support for self-management. Healthcare managers were interviewed to understand the structure of T2D care and to understand patient’s perceptions of pathways of care starting from primary care to tertiary care or other services.
	Community stakeholders/health actors (including: community members or group leaders active in formal and informal groups)	13	7	Participants were recruited from socioeconomically disadvantaged communities through snowballing with support from a community member who helped in accessing the community groups.	- 4 Group interviews were conducted with members active in language classes, and Iraqi and Turkish associations. 14 individual interviews were held with group leaders or members active in informal groups, local shop owners and sports/gym managers. The interviews were conducted in Swedish and an interpreter was used if participants preferred their native language. All participants signed an informed consent form prior to participation. Interviews were conducted in Swedish by two research team members lasting 30–60 minutes. Participants received two movie tickets for participation. The information power from the study sample was sufficient to address the research questions [27].	- The main themes in the individual/group interview guides included: Perceptions of community, community, Perceptions of health and care and support for persons with diabetes within the community.
	Relevant local businesses (including: sports/gym manager and local shop owners)	2		- Local business owners were included during fieldwork by one researcher based on their availability and proximity to the study setting.		
	Stakeholders at local/municipality and regional level (such as development strategist, social worker, Swedish language teacher,—health educator from the County council etc.)	4	4	- The stakeholders were purposively sampled from socioeconomically disadvantaged communities to include representatives of local and regional institutions working with welfare, public health, social and economic aspects, self-management, and those frequently meeting immigrants in their daily work to understand their experiences and strategies of engaging with socio-economically disadvantaged communities in the prevention and management of T2D.	- 8 Individual interviews were conducted with representatives from the local government, local NGOs and regional institutions (including; the municipalities and the county council). All participants signed an informed consent form prior to participation in the interviews. Interviews were conducted in Swedish by two research team members lasting 30–60 minutes. Participants were given two movie tickets for participation. The study sample provided sufficient information power to address the research questions [27].	- The main themes in the guide included: Organizational responsibilities, interactions with the community, awareness of diabetes burden in the community and health promotion and diabetes prevention.
	Health system	5 primary healthcare centers		- The healthcare centers were purposively sampled to include centers located in socioeconomically disadvantaged communities and their involvement in a diabetes the screening program and their interest to participate in the study.	- 5 observations of care practices and processes at primary health centers were conducted by one of the research team members who had prolonged engagement in the community.	- The observations were based on the following themes: Existing pathway for diabetes care at the primary healthcare center and existing strategies and referral systems.
	Physical environment	5 communities within the study setting		- The communities were purposively sampled to include socio-economic suburbs where the study participants resided.	- The activities within the community networks, food and physical activity structures were observed during the period of data collection.	- The observations of the physical environment focused on: Existing community activities, networks/groups, food and physical activity structures
**South Africa**	People diagnosed with T2D	18	22	- Participants diagnosed with T2D/at risk of T2D were recruited from the PURE database[29] and the health facility by health providers in the participating facilities. Interested participants were approached by the research team to schedule interviews. People at risk of T2D were recruited from a health club attached to the facility or from their community.	- 5 Focus Group discussions (FGDs) were held with men and women aged 30–72 years, diagnosed with T2D and 2 with persons with known risk of T2D All participants signed an informed consent form prior to the interviews. The FGDs were conducted in Xhosa by two members of the research team lasting between 45 and 90 minutes. The FGDs were conducted till saturation. The participants received a transport facilitation of +/- US$ 3,5 and refreshments. Further details of participant recruitment and data collection process are published elsewhere [30].	- main themes were: Illness perceptions, health seeking behavior/ practices; lifestyle risk factors (diet / PA) and community support; health education needs related to T2D
	People at risk of T2D	8	8			. The main themes for the participants at risk included: Risk perceptions, health seeking behavior / practices; lifestyle risk factors (diet / PA) and community support; health education needs for persons at risk of T2D.
	Health care providers: - Physician - Nurses in charge of the facility-based health club		3	health providers involved in the care/management of T2D patients from the participating health centers.	- 3 In-depth interviews were conducted with nurses in charge of the health club; and medical practitioners involved in the management of the T2D patients. Informed consent was obtained from the providers prior to participation. The interviews were conducted in English by two research members lasting between 45 and 90 minutes. Interviews were conducted till saturation.	- The interview guide for the providers included the following themes: Profile of T2D patients, screening and type of care provided, availability of community support; defaulter tracing and follow-up, dealing with co-morbidities and use of traditional medicine.
	Community stakeholders/health actors including: Diabetes SA Western Cape Branch Manager MSF HIV/AIDS patient adherence and community support manager Caring Network Director		3	- The community stakeholders were purposively sampled based on their responsibilities and involvement in community-based programs.	- 3 Informal discussions were conducted with key informants from civil society including the Diabetes SA Western Cape Branch Manager. Three observations of activities in the existing community T2D support groups was also conducted. Informed consent was sought prior to the interviews. The interviews were conducted in Xhosa lasting between 45 and 90 minutes. Interviews were conducted till saturation.	- The interview guide included questions related to activities in the community based support clubs and lessons learned.
	Health System - Director of local health services	1	5	- Patients visiting the participating health center were conveniently sampled for observation and key informants including the manager of health services was approached for interviews.	- Observations of the care practices and processes at primary health centers were conducted and one in-depth interview was held with the director of local services.	- The observation and interview guide focused on pathways of T2D care and referral process, and government programs in place.
	Physical environment	139 households		The households were systematically sampled.	- Community members were observed during their visits to the health facility and 250 questionnaire surveys were conducted with community members regarding the food environment.	- The observations focused on: Existing community activities, networks/groups, food and physical activity structures. The questionnaire explored participants’ dietary choices and patterns in the households of the study area, and impact of these choices on the risk factors for diet-related NCDs.
**Uganda**	People diagnosed with T2D	25	25	- People diagnosed with T2D were identified from the patient database by the doctors at the health center. The participants were purposively sampled to include men and women with T2D actively receiving care at the participating health center.	- In total 16 FGDs and 8 in-depth interviews were conducted with men and women diagnosed with diabetes and those at risk of T2D aged 35–60 years. Informed consent was sought from all the participants involved. The Interviews/FGDs were conducted in Luganda by the two research members lasting between 45 and 90 minutes. The participants received a transport facilitation of +/- US$ 1,35 and refreshments. All interviews and FGDs were conducted till saturation. Further details of participant recruitment and data collection process are published elsewhere [31].	- The themes explored included: Illness perception and health seeking behavior / practices; lifestyle risk factors (diet / PA) and community support; health education needs related to T2D.
	People at risk of T2D	25	25	- Adult patients receiving care at the participating health centers’ out-patient department were purposively sampled from the patient database at the facility based on the following criteria: a known history of hypertension and/or on medication for hypertension and being overweight with BMI>25kg/m^2^.		- Main themes including: Risk perceptions, health seeking behavior / practices; lifestyle risk factors (diet / PA) and community support; health education needs related to T2D.
	Health providers - MOH Key Informants, district health officer, district health educator, clinical officers, nurses, nursing aids, village health team members	5	10	The providers were purposively sampled based on their responsibilities and involvement in NCDs control activities at the MOH central level or local district health service level and health care delivery at the primary healthcare level.	- In total 15 In-depth interviews were conducted with providers from each level of the public healthcare system including: MOH Key Informants, district health officers, district health educator, 2 clinical officers, 2 nurses, 2 nursing aids, 2 village health team members. The participants signed an informed consent form prior to the interviews. The participants received a transport facilitation of +/- US$ 1,35 and refreshments. Interviews were conducted in Luganda by two members of the research team lasting between 45 and 90 minutes. The interviews were conducted till saturation.	- the following themes were explored: Type of care provided that is relevant to type 2 diabetes care and prevention; status of the minimum package of diabetes services in assessed health facilities; availability of equipment and drugs for diabetes and associated risk factors; and support services for people with risk factors and with diabetes.
	Community Stakeholders/health actors	8	11	The participants were identified from the villages within the study area with the help of local leaders based on the following criteria: Residents of the villages, responsibility and involvement in Chronic disease management/care within the areas.	- 4 FGDs with community members living in the study area and 4 In-depth interviews with stakeholders of organizations involved in HIV care were conducted. 4 in-depth interviews were held with stakeholders of organizations involved in other chronic diseases care. Informed consent was sought from all participants. The participants received a transport facilitation of +/- US$ 1,35 and refreshments. The interviews and FGDs were conducted in Luganda by two team members lasting between 45 and 90 minutes. The interviews/FGDs were conducted till saturation.	Interview/FGD guides included topics on: Illness perception and health seeking behavior / practices; lifestyle risk factors (diet / PA) and community support; health education needs related to T2DM.
	Health System			- Health facilities were purposively-selected to include facilities from the primary and secondary care levels of the public health care system.	- Observations of care practices and processes at primary health care centres and MOH records (2 hospitals, 1 HC IV, 2 HC III, 2 HC II) were conducted.	- The observations were focused on: Availability of chronic care services, equipment, drugs and sundries to support chronic care and prevention of type 2 diabetes and related conditions and how health facilities cope with non-availability of essential supplies for chronic care.
	Physical environment			The villages within the study areas were purposively selected to participate.	- Observations of the activities within the community networks, food and physical activity structures were conducted.	- The observations included: Existing community activities, networks/groups, food and physical activity structures.

K = 165 the number of groups, MOH = ministry of health, T2D = type 2 diabetes, PURE = Prospective Urban and Rural Epidemiological cohort, 4D = 4 Diagnoses project, HC = Health Centre, BMI = Body Mass Index

### Data-analysis

Data-analysis was informed by the framework method which allowed exploring data systematically and in-depth, while maintaining an effective and transparent audit trail and facilitating collaboration among our multidisciplinary team [32]. The analysis followed four steps: (1) Theoretical coding of the raw site-specific data by each country research team: Site-specific analysis of the data sets was conducted by multidisciplinary teams of 5–7 members in the respective sites who comprised: health systems researchers including medical doctors and nutritionists, public health scientists, intervention and implementation research experts, and anthropologists. Three research team members in each of the sites coded the data using NVivo software version 11 in Sweden and Ti software version 7.0 in South Africa and Uganda. Categorizing of similar codes into themes, assessment and refinement of the final themes and sub-themes was collectively done by the respective site teams. Some of the site-specific data is published elsewhere [28,30] and others are under review; (2) For the purpose of cross-site data synthesis and this paper, the site-specific data were assigned to a set of themes predefined and organized based on the structure in the cross-site topic guide. Data was triangulated from different sources including interviews, observations and literature, resulting in a country-case description; (3) Data from three sites was then systematically charted using a framework matrix (see Table 2 of the results section) following the main topics of the framework: the individual, the individual mediators, family and friends, the health providers, the community health actors, the health system, the social environment, and the physical environment; (4) The elements identified in the previous steps were classified as ‘differences’ or ‘similarities’ between sites. This classification was based on the element’s presence and estimated contribution to self-management in a particular context. An element meeting those two criteria in one context and not in another context was classified as a difference. If it met both criteria in two different contexts, it was classified as a similarity. Two experts (JDM, JVO) evaluated each element based on those two criteria. In case of disagreement, the element was discussed with a third expert (MD). Results were then shared with the specific country teams and in case of disagreement, the elements were discussed a third time.

### Ethics approval

The study was approved by the ethics committees in each of the respective countries. In Uganda, this was the Higher Degrees, Research and Ethics Committee (HDREC) of Makerere University School of Public Health and the Uganda National Council for Science and Technology (Ref. HDREC-331 and HS 1917 respectively), in South Africa this was the Senate Research Committee of the University of the Western Cape (Ref. 15/3/17), in Sweden, this was the Regional Ethics Review Board in Stockholm (Ref. 2015/712-31/1), and in Belgium, the Institutional Review Board (ref 993/14).

## Results

### Results of the site-specific analysis

Table 2 presents the results by site (vertically) and by main- and sub-topics (horizontally) following the structure of the topic guide.

**Table 2:** Results of site-specific analysis.

Framework element	Uganda	South Africa	Sweden
**The Individual:**			
Characteristics of the general population in the study area	- Low education levels (literacy approx. 46%), socio-economically disadvantaged, and poor housing conditions [SD] - About 60% of the population involved in subsistence agriculture, mostly using hand hoes [SD]	- Socio-economically disadvantaged population: very low income, poor housing conditions, and low education (some are illiterate) [SD] - Mainly black population (>99%). Diversity in languages, but mainly Xhosa speaking (>90%) [SD]	- Socio-economically disadvantaged compared to other districts in Stockholm County: lower employment and income levels, poorer housing conditions, lower education levels, lower social mobility, and more limited Swedish fluency [SD] - High proportion of migrants (mainly former Yugoslavia, Somalia, and Turkey) with diversity in culture and ethnic background [SD]
Mobility	-Stable population, low levels of migration [SD], [CI] - Majority live with their family members [II], [CI]	- Frequent moving (to visit family or for work purposes) hinders continuity of care [II], [CI] - Many relocated to this township for work, leaving the rest of their family behind in rural areas [II], [CI]	- Frequent moving of target population hinders continuity of care [PI] - The majority of the population live with their family [II], [CI]
Disease burden	- High prevalence of acute and chronic infectious diseases [SD]	- High prevalence of chronic infectious and non-communicable diseases [II] [SD]	- Disproportionately affected by chronic non-communicable conditions [SD]
**Individual Mediators:**			
Perceived autonomy	- Limited pro-activity of patients during consultations with providers [PI] suggests low perceived autonomy. Pro-activity increases among patients who manage their illness for a longer time [PI].	Lack of perceived autonomy support for dealing with T2D care and treatment [30]	- Individuals feel that they are not given the opportunity to express their challenges/concerns during consultations with providers [II]. This suggests low perceived autonomy.
Perceived relatedness	- Individuals report to receive support from family and friends [II], suggesting perceived relatedness	- Low perceived relatedness regarding health care providers [30]	- Individuals report to receive support from family in their self-management which suggests perceived relatedness [II].
Self-efficacy	- Reported barriers under physical and socio-cultural environment (see below) [II] suggest low self-efficacy.	- Reported barriers under physical and socio-cultural environment (see below) [II] suggest low self-efficacy. - Patients experience a lack of self-efficacy to effective self-management [30]	- Reported barriers under physical and socio-cultural environment (cfr. below) suggest low self-efficacy [II].
Illness representation	- Awareness of common causes and risk factors of T2D (e.g. obesity, sedentary lifestyle) [II], [PI] - Misconceptions generated through traditional and religious beliefs [PI] - T2D is perceived as dangerous [II] - Reluctance to seek care for early signs and difficulties to adhere to treatment in absence of symptoms [PI]	- Awareness of common causes and risk factors of T2D [II] - Misconceptions generated through traditional and religious beliefs [II], [PI] - T2D is perceived as severe [II] - Acute symptoms that directly affect people (e.g. headache) are stronger triggers of health seeking behavior than symptoms that may indicate an underlying disease like T2D but don’t affect people directly [II]	- Awareness of common causes and risk factors of T2D [II], [PI] - T2D is perceived as a common disease and part of the aging process, depending on one’s genetic profile. Some do not perceive T2D as severe [PI], [II] - Some individuals have frustrations because of not seeing any changes in clinical parameters after doing efforts to change their lifestyle [II] - Some patients who don’t feel sick don’t see the need for self-management [PI], [II] - Some doubt if lifestyle change can prevent T2D [II]
Learning of self-management strategies	- Awareness of the beneficial effect and the meaning of a healthy diet, physical activity, and routine check-ups [II] - Traditional and religious beliefs lead to misconceptions [PI]	- Awareness of maintaining a healthy diet and doing physical activity [II] - Traditional and religious beliefs lead to misconceptions [PI]	- Awareness of the recommendations regarding lifestyle and self-care, but difficulties to translate these to their particular situation [II] - Holding on to traditional or cultural beliefs interferes with recommended treatment [II], [PI]
**Family and Friends:**			
Psychological support	- Family and friends provide emotional support [II] - Family members provide support in treatment [II]	- Family and friends provide psychological support [30]	- Family and friends inspire and motivate patients to adopt and integrate lifestyle changes into their daily life [II] - Family and friends trigger individuals to seek health care [II] - Some patients perceive illness as a private problem, which they only share with close family [II] - Limited expectations of support from friends and peers because health is seen as something personal [II]
Practical support	- Family members provide support in domestic tasks [II]	No Data	- Family members help in preparing meals [II] - Family members help by accompanying to clinic visits [II]
**Health Providers:**			
Consultation time	- On average, consultation time is short [OH]	- On average, consultation time is short. Due to different dialects, language can be a barrier [OH], [PI]	- Short consultation time and language are reported as barriers to communication [OH], [PI]
Orientation to care	- Providers have a biomedical orientation with little attention for patient preferences or psychosocial background. At the private hospital, providers give more attention to individual context and preferences [OH], [PI]	- Biomedical approach, with some attention for patient preferences and psychosocial aspects [OH], [PI]	- Taking into account the psychosocial context remains a challenge for providers, although they acknowledge its importance [II], [PI]
Patient involvement	- Providers approach is usually directive with no or minimal patient involvement [OH] - More involvement when patients have been managing their condition for a longer time [OH], [PI]	- Some providers are open to involved decision making, but time is a constraint [PI]	- Variation among providers in how much they attempt to stimulate involved decision making[OH], [PI] - Patients lacking pro-activity and consultation time constrain patient involvement [PI]
Self-management education	- Self-management education is limited and not tailored [II], [PI] - Traditional and poorly trained practitioners often provide misinformation [PI] - Providers stimulate patients to link up with a self-appointed treatment supporter [PI]	- Self-management education is limited because of overcrowding at the health center [OH] - Traditional and poorly trained practitioners often provide misinformation [PI]	- Patients are not provided with information on how to integrate lifestyle changes into daily life [II] - Providers are not adequately equipped to deal with the diverse cultural needs of their patients [PI] - in 50% of the health centers, designated T2D nurses provide self-management education [PI]
Health promotion activities	- Patient are referred to a diabetes club for health promotion and medication counseling; the club is localized at the hospital and led by peers [OH], [PI]	- Health centers work with diabetes clubs localized within the health center: stabilized patients (acceptable glycated hemoglobin and medication adherence) are referred to this club for follow-up which includes health promotion, medication counseling and follow-up of parameters [OH], [PI]	- No regular joint activities organized for patients [PI] - Sporadically, health centers organize health promotion activities like accompanied walks [PI]
**Community Health Actors:**			
	- No community initiatives relevant to self-management identified [CI]	- A variety of NGO driven support groups in the community organize different activities like screening, treatment follow-up, and exercise promotion [CI]	- Some NGOs organize health promotion sessions [PI], [CI] - The municipality organizes ‘ad hoc’ specific consultations to inform migrants and asylum seekers about health, including T2D [CI]
**The Health System:**			
Type of health system and providers	- Mixed public-private system; private and/or traditional practitioners & pharmacies respond to people’s unmet demand [OH], [PI], [SD]	- Mixed public-private system with first-line care offered by public health centers, informal and private providers and pharmacies [OH], [PI], [SD]	- Public funded system with first-line care offered by public and private providers [OH], [PI], [SD]
Health care staff capacity	- Poorly qualified staff with a lack of training in T2D care at primary health centers. At referral level: weekly diabetes clinic, run by medical officers trained in T2D care [OH], [PI], [SD], [31]	- Primary health centers have qualified staff with training in T2D. Specialized staff is available at the referral hospital [OH], [PI], [SD]	- Well trained and qualified staff, designated T2D nurses for self-management education in 50% of health centers [OH], [PI], [SD], [28]
Guidelines	No accurate guidelines are available [OH], [PI], [SD]	Guidelines available for T2D treatment, not for prevention [OH], [PI], [SD]	Up-to-date and evidence-based guidelines for treatment and prevention, but no guidelines /training on culturally adapted lifestyle support [OH], [PI], [SD]
Type of care	- T2D care is cure oriented and with little attention to health promotion, prevention, and rehabilitation [OH], [PI]	- TD2M care includes basic health promotion, prevention, and rehabilitation [OH], [PI], [SD]	- T2D care includes health promotion, prevention, diagnosis and treatment, to rehabilitation, palliative care, and social services [OH], [PI], [SD], [28]
Access to care	- Difficult geographical access to the formal health system. First-line T2D care only available at the referral centers. No formal user fees at public facilities. Oral and anti-diabetic drugs, insulin and basic lab-tests (no HBA1C) offered without user fee, but stock-outs are frequent. Only about 5% of patients can afford a personal glucometer [OH], [PI], [SD], [II], [31]	- Public services are geographically accessible, but daily queues are long. First-line T2D care offered free of cost at primary public services; including essential medication [OH], [PI], [SD], [II]	- Good geographical access to care, but long waiting times (to get an appointment) can be a barrier. Medication and consultations available at relatively low cost (pre-determined co-payment with ceiling). Prescribed insulin and self-management tools like glucometers are free of cost [OH], [PI], [SD], [II]
Continuity of information and coordination of care	Patients carry their own medical file. Very limited communication/coordination between different levels of care [OH], [PI], [SD]	Communication and coordination over different levels (hospital-health center) is limited. Health centers keep a paper-based medical file of patients in follow-up. Providers can access lab-tests done in other locations of the country, which contributes to continuity of care [OH], [PI], [SD]	- Multi-disciplinary team approach, adequate referral system, and electronic health records contribute to integrated care [OH], [PI], [SD], [28]
Interactions between the health system and the community	Not applicable because no relevant community initiatives identified	- Providers refer patients to community-based service providers. [OH], [PI], [II] - The formal health system distributes medication through community-based initiatives. [OH], [PI]	No formal interactions. Providers may give lectures in the community during “health days” organized by some of the healthcare centers [PI]
**Socio-cultural environment:**			
Community ties	- Strong community ties [SD], [II]	- People respect their community, but frequent migration hinders a development of strong community ties [30], [II]	- Community ties are perceived as weak [II], [CI] - Gatherings among community members are often religiously or socially inspired, and often, but not exclusively, based on descent [II], [CI]
Social stigma	- Limited stigmatization of persons with T2D, unless severe complication like a diabetic foot [II], [PI]	- T2D may be linked to a bad lifestyle and sufferers blamed for their obesity and lack of physical activity [CI]	- Stigmatization of persons with T2D is limited, but mentioned as a potential barrier to seek treatment [II], [CI]
Attitude towards obesity	- Obesity is a sign of being wealthy for some [II], [PI]	- Obesity can be stigmatizing as greediness but also a sign of success and ‘having a good life’ [II], [CI]	No data
Attitude towards physical activity	- Doing sports to improve your health is perceived as strange [II], [PI]	- The idea of doing sports is poorly adopted among the older generation [II]; walking and physical labor represented hardship in the past [30] - Lack of willingness and resources to exercise [II] - Exercise associated with health and spiritual benefits [II]	- The idea of doing sports to improve your health is poorly adopted in the target population [II], [CI] - Women going to mixed gyms is not accepted in certain ethnic/religious groups [II], [CI]
Dietary customs	- Traditional diet is rich in carbohydrates, but also includes fruits and vegetables [II], [OP]	- Availability, convenience and preference for less healthy foods [II]	No data
**Physical environment:**			
Barriers to physical activity	- Weather conditions and perceived lack of safety [II]	- Weather conditions and perceived lack of safety [II]	- Weather conditions and perceived lack of safety [II]
Sports facilities	- Outdoor sports fields are present [OP]	- Outdoor sport facilities are present, but mainly used by adolescents and young adults. Indoor sport facilities are deemed expensive [OP], [II]	- Neighborhoods offer opportunities for outdoor sports [OP] - For indoor sport facilities, the user fee is mentioned as a barrier [II]
Access to un/healthy food	- Increasing access to refined flour and cooking oil [OP], [II]	- Healthy and unhealthy food is available, but individuals perceive unhealthy food as more accessible and convenient compared to healthy food [II]	- Easy access to both healthy and unhealthy food, although healthy food is generally perceived more expensive and unhealthy food more convenient [OP], [II]

Where relevant, the main source of information is provided within brackets as follows: Interviews with individuals with or at risk of T2D [II]; Provider Interviews [PI]; Interviews with community stakeholders [CI]; observations of the health system [OH]; observations of the physical environment [OP]; and secondary data, such as national statistics, other studies and project documents [SD] or [ref.]. T2D = type 2 diabetes.

### Cross-comparison

The sites share characteristics in terms of awareness of risks, knowledge about self-management strategies, perceived relatedness, and barriers to implementing these strategies such as a lack of perceived autonomy and self-efficacy. Quality of interaction between providers and people at risk of T2D (i.e. interpersonal quality of care) has room for improvement in the three sites. Most obvious gaps are the lack of a tailored approach and the lack of patient involvement. Participants across the three settings mention failing to implement self-management in their daily routines and perceive a need for more tailored self-management education. The sites share similarities in determinants related to the physical environment (e.g. perceived barriers to physical activity or a healthy diet).

The most noticeable differences across sites relate to structural quality of health service delivery (i.e. accessibility, technical quality of care, continuity of care) and the presence of community initiatives. Quality of health service delivery is very high at the Swedish site, meets essential standards at the South African site, and is poor at the Ugandan site. Presence of self-management-related community initiatives for T2D is high at the South African site, limited at the Swedish site, and absent at the Ugandan site. The ways that people perceive the socio-cultural environment (attitudes, norms, and values) as influential on their lifestyle can be very similar (e.g. limited to no stigmatization of people living with T2D) or very different across sites (e.g. obesity as a sign of wealth in South Africa compared to Sweden).

Table 3 presents the main differences and similarities across sites in more detail per main topic of the topic guide.

**Table 3:** Differences and Similarities among countries.

Framework topics	Similarities	Differences
Individual: characteristics of the study-population	- Low socio-economic status - Relatively high prevalence of chronic diseases	- SWE & SA: High proportion of migrants, frequently moving - UG: Mostly native population
Individual Mediators	- Indications of perceived relatedness, but low perceived autonomy and low self-efficacy - Individuals show awareness of common causes of T2D and major self-management strategies. - Religious and traditional beliefs influence people’s knowledge.	- UG & SA: perception of T2D as severe and dangerous. Acute symptoms were reported as important triggers for health care seeking, in contrast with symptoms that may indicate a risk but do not directly affect people. - SWE: people describe T2D as a common illness and some question the use of lifestyle management to prevent T2D because of the genetic disposition.
Family and friends	Provide psychological support	- UG & SWE: provide practical support at home
Health providers	Interactions with patients fall short in at least two key aspects: a tailored approach and patient involvement, especially at the Ugandan and South- African site.	- UG: providers systematically stimulate patients to link up with a self-appointed treatment supporter. - UG & SA: stable patients are referred to a diabetes club within the health facility, for health promotion and self-management education. The club in Uganda is led by an expert patient, the one in South Africa by a nurse, who also does routine measurements. - UG & SA: Traditional and poorly trained providers provide misinformation to patients.
Community health actors	Community initiatives differ strongly in scope and purpose in each site.	- SA: A variety of NGOs organize community activities such as self-management education and distribution of medication. - SWE: community initiatives were less prominent and included initiatives from the municipality and NGO’s. - UG: Community initiatives supporting T2D self-management were not identified although other health related community initiatives exist.
Health system	Prominent themes influencing self-management are the quality of first-line care, geographical, and financial accessibility.	First line health care for T2D differs strongly in the three settings. - UG: a lack of supplies, qualified staff, and guidelines, hamper quality of care and geographical and financial access. - SA: essential diabetes care including prevention is accessible and free at primary level, although waiting times may be long. - SWE: integrated care with multidisciplinary teams, referral systems and electronic medical records, but reported waiting times and copayments may discourage patients.
Socio-cultural environment	- Stigmatization of people with T2D is limited - Respondents mention that sport is not commonly considered as a way to improve health	UG: people are well rooted in their communities SA: community ties are less strong because of migration SWE: community ties are very loose - SA & UG: obesity can be seen as a sign of success
Physical environment	Similar perceptions on barriers to physical activity (e.g. weather conditions, perceived lack of safety)	SA & SWE: people mention unhealthy food to be more accessible and convenient than healthy food.

UG = Uganda, SA = South Africa, SWE = Sweden, T2D = type 2 diabetes

## Discussion

To our knowledge, this is the first study to explore self-management determinants of T2D among disadvantaged populations in three different settings through the use of a common guiding framework. Earlier studies on disadvantaged populations confirm the influence of psychological factors (e.g. knowledge, beliefs, behavioral skills, etc.)[33] and the individual’s socio-cultural context, including social support networks [34,35], and motivational support from health care providers [36]. However, those studies have not compared different contexts and had a narrow focus on a specific set of elements. Our data cover a comprehensive set of elements that play a role in the implementation of self-management including the individual and their family, health- and community actors, the health system, and the proximal environment. The study links these elements with self-management behavior through individual mediators.

The most noticeable differences across the sites relate to structural quality of health service delivery and the presence of community initiatives. The health system in Uganda is characterized by inadequate basic supplies, shortage of qualified staff, and lack of guidelines, whereas in South Africa, essential diabetes care including secondary prevention is accessible and free at primary level. In Sweden, primary care is more advanced involving multidisciplinary teams, referral systems and electronic medical records, but is primarily facility based and has limited activities focusing on prevention. These differences are linked to the macro-economic status of the country and the historical development of the respective health systems. In SMART2D, we used these inherent differences in the choice of our sites to inform the development of a contextualized self-management support intervention and to learn from each other during this process. The role of the community in diabetes related health promotion and prevention and the linkage between community and the health system is stronger in South Africa than in the other two settings. We identified several elements playing a role in self-management related to people’s proximal environment, mostly relating to lifestyle behavior. Similar to other studies, this demonstrates the importance of the physical and socio-cultural environment on lifestyle behavior [37,38] Social and cultural factors influencing people’s lifestyle were more similar between Uganda and South Africa and different from the Swedish setting.

Across the three study sites, participants are aware of the risks of T2D and recommended self-management practices. However, as reported by other studies, integrating those practices into their daily life is challenging [28,39]. Our data identify multiple interrelated factors that may explain this limited integration. Study participants across all three sites share low levels of perceived autonomy and self-efficacy which could be partially explained by patient-provider interactions with limited patient involvement, low autonomy support of patients, and a lack of tailored education. In all sites, participants reported receiving psychological support from friends or family, suggesting perceived relatedness.

This lack of self-efficacy and perceived autonomy may hinder implementation of self-management even if the structural quality of the provided care is excellent as is the case in Sweden (see below). Addressing the lack of structural quality in, for example, the Ugandan setting may therefore not lead to the desired improvement of self-management if such an intervention does not address the reported low self-efficacy and low perceived autonomy among people living with T2D. Similarly, the reported awareness of major self-management strategies suggests no need to increase self-management education, but rather to reconsider the way education is being implemented, i.e. based on active learning and with more attention for mediators like self-efficacy, perceived autonomy, and while addressing misconceptions with regards to traditional beliefs.

### Methodological considerations

To our knowledge, the transdisciplinary framework presented in this study was the first to combine a comprehensive set of elements that determine self-management (actors, the health system, the environment, etc.) with individual mediators from a behavior change perspective. This approach allows for an explanation of how external elements (e.g. the health system, the proximal environment) influence individual mediators and ultimately self-management behavior within a real-life context and addresses limitations in the understanding of self-management implementation. The strength of the proposed framework lies in the use of generic pathways that link the proximal environment and the health system with the individual’s behavior or self-management for different settings enabling cross-comparison. This contributes to a better understanding of self-management, beyond the traditional explanations from a health systems perspective (e.g. a lack of resources) or a population perspective (e.g. low socio-economic status). Consequently, this framework also identifies other and eventually more feasible solutions beyond the traditional structural changes (e.g. improve the infrastructure of the health system). Bronfenbrenner’s socio-ecological framework, which has been widely used in public health, also addresses the determinants of health at different levels. However, it does not account for the pathways explaining the individual’s behavior [40]. Bronfenbrenner attempts to explain this at cognitive level through ‘force characteristics’, nevertheless those characteristics seem to be a collection of personal traits and cognitive concepts (e.g. temperament, self-efficacy, etc.) without a clear link between the environment and one’s behavior [41]. Brown et al. also linked individual and external factors influencing self-management, but mainly focused on elements related to the individual’s socioeconomic position, ignoring individual psychological mediators [42]. Berkman et al. highlighted the importance of psychological pathways by linking social integration with health, but their study discussed health in general and was not focused on self-management or chronic diseases [43].

The study methods facilitated cross-learning among different sites through the use of a common conceptual framework and the framework method. The active involvement of the local research teams in the translation of the framework to the data collection guide facilitated contextualization. The framework was comprehensive and yielded rich data on the determinants of self-management in the respective contexts, but translation of the framework concepts to data collection tools was difficult and resource intensive. The process required several online meetings and workshops with the implementation teams from the respective study sites. Translation of the framework required a focus on certain elements at the cost of others, based on what the teams estimated as relevant for their context and what was feasible in terms of data collection.

Application of the framework for data collection was equally challenging for the implementation teams. They perceived the topic guide as very broad, theory-driven, and difficult to adapt to the three local contexts. Abstract concepts like the psychological mediators (e.g. perceived autonomy, self-efficacy) were difficult to translate and measure, which could explain why data related to some of these theoretical constructs is sparse for all study sites. This sparseness of data hindered the full application of the framework and as a consequence, the understanding of self-management. Actual data collection was also hindered by factors like limited human resources, a lack of security for data collection teams in some study areas, particularly in South Africa, and delays in mobilizing community stakeholders and healthcare providers, particularly in Sweden.

The major part of our findings resulted from a triangulation of interviews with different participants or other sources of information (e.g. observations, evidence from the literature), which contributed to the credibility of the results. Findings relate to populations which are disadvantaged in similar ways (socio-economically, educationally, type of housing, etc.), but are living in three different settings. Compiling evidence based on data from three different sites contributes to the transferability regarding disadvantaged populations of similar LMICs in Sub-Saharan Africa, and most likely also in HICs.

Cross-comparison of the different sites led to useful insights on how different environments can contribute to self-management through similar pathways. As illustrated before: addressing environmental or health system related shortcomings, while ignoring those mediators, may therefore not lead to the desired effect. The authors acknowledge that the sparseness of data regarding those mediators has hindered cross-comparison. More precise information regarding those mediators may therefore lead to a better understanding of how different contexts or environments and related interventions influence self-management.

## Conclusion

The implementation of self-management relates to a complex interplay between the individual, the socio-cultural and physical environment, the health system, and related actors. Implementing self-management in a particular context will benefit from an overarching framework contextualized through a situation analysis. Essential is that such a framework not only identifies the necessary self-management support interventions, but also how these interventions need to be implemented. This can be obtained through consideration of the pathways linking the individual’s behavior with its proximal environment.

This study uses a transdisciplinary framework to identify major gaps and opportunities to guide the implementation of self-management support in low-resourced or socially disadvantaged areas and populations compiling evidence from three different settings.

Findings indicate that while the studied populations are aware of what self-management for T2D entails, the integration in their daily life is difficult. Despite being in completely different settings, individual mediators and perceptions of the physical (built) environment determining self-management are similar in the three disadvantaged populations, while health systems determinants and community support for self-management largely differ among sites. Depending on the setting, opportunities to facilitate implementation of self-management include: making patient-provider interactions more person-centered, improving access to essential primary care, and encouraging community initiatives supporting self-management. The individual’s physical environment (e.g. accessibility of healthy food) and socio-cultural environment (i.e. norms, values, and social support) play an important role in people’s lifestyle and offer opportunities for change.

The SMART2D self-management framework was developed based on literature reviews and expert consultations, and applied in this study to inform data collection, analysis and interpretation. However, to assess the internal validity and interconnections between different elements, quantitative research is needed. The findings of the present study set a point of departure for research that seeks to understand the pathways for implementation of self-management support interventions. The identified gaps and opportunities can be addressed in field trials focusing on the development and implementation aspects of self-management interventions. The findings from this study may be applicable to disadvantaged populations in similar sub-Saharan LMICs and HICs with vulnerable populations.

## Supporting information

S1 Appendix Site-specific interview and FGD guides. (PDF)

S2 Appendix Generic topic guide. (DOCX)
