# Supplementary material for: Diabetes self-management in three different income settings: Cross-learning of barriers and opportunities
Source: PLoS One. 2019 Mar 19;14(3):e0213530. doi: 10.1371/journal.pone.0213530 (PMC6424475; doi:10.1371/journal.pone.0213530)
Supplement: S1 Appendix — (PDF) [file pone.0213530.s001.pdf]

Supplementary file 1:
Site specific interview and FGD guides

Interview/FGD guides: Uganda

Semi-structured interview/FGD guide for patients with type diabetes

Theme 1: Notions of well being

1. What do you consider as 'well-being' in terms of health?
2. Do you think it is possible for a person with diabetes to have 'wellbeing' the way you have described it? Explain why or why not
3. Do you think that future well-being matters in a person's life? Explain why or why not?

Theme 2: Health seeking behavior

4. In what situations is it 'normal' for a person to go to a health center to seek care?
5. In your opinion what do you think about people visiting a facility when they are feeling well?

Theme 3: Diet and dietary behavior

6. What do people generally eat in this community (Habitual diet)?
7. Doctors recommend a balanced diet. How do you describe a balanced diet?
8. What is the importance of eating a balanced diet?
9. Are there any challenges in practicing the doctor's advice on a balanced diet?
10. Can you tell us the food items people with diabetes should not eat?
11. Can you describe to me what you understand as a bad diet?

Theme 4: Facilitators of eating a balanced diet

12. Despite the barriers you have mentioned above, how can we encourage people to eat a balanced diet?
13. In terms of culture, what are the community beliefs regarding eating the food items that form a balanced diet?
14. In general, how do people prepared food in this community?

Theme 5: Physical activity

15. What is your understanding of physical activity (any body movement that results in expending energy)?
16. Is regular physical activity important for living a healthy life in the present and future?
17. What are the different physical activities that you take part in from morning to evening?
18. In case you need to meet the recommended level, what do you think could be the acceptable physical activities for the following?
19. In case you need to do more to meet the recommended level, what do you think could stand in your way of meeting and maintaining this goal?
20. What do you think could make it easier for you to meet and maintain the recommended level?
21. When do you think that it is necessary for a person to engage in physical activity?

Theme 6: Experiences with care

22. What are your opinions regarding reaching this health facility where you get your regular treatment for type 2 diabetes?
23. Let's talk about the services you get from this health facility, what kind of services in regard to diabetes treatment do you get when you reach this health facility?
24. In your opinion do all patient get these services?
25. What do you have to say about the quality of services you have mentioned?
26. How do the healthcare providers involve you in deciding about the treatment you get?
27. How have the health workers empowered you to manage your disease?
28. What kind of support do you receive from the health workers, CHWs, community and family in managing your disease?
29. How are people with diabetes being motivated to change their behavior in managing the disease in your community?
30. What are the avenues available to address patient grievances and complaints?

Theme 7: Risk communication

31. Is raising awareness about diabetes in the communities important?
32. Through what communication channel do you learn about matters of health?
33. Which of the communication channel is most preferable to you at individual and community levels?
34. You receive lifestyle education whenever you visit the health facility. What do you think about the information you have received?

Semi-structured interview/FGD guide for person at risk type 2 diabetes

Theme 1: Notions of well being

1. What do you consider as 'well-being' in terms of health?
2. Do you think it is possible for a person with diabetes to have 'wellbeing' the way you have described it?
3. Do you think that future well-being matters in a person's life? Explain why or why not?
4. Does the fact that you know that you are (over-weight/have high blood pressure) have an effect on your well-being?

Theme 2: Health seeking behavior

5. In what situations is it 'normal' for a person to go to a health center to seek care?
6. In your opinion what do you think about people visiting a facility when they are feeling well (or when they have no complaint of sickness at all)?

Theme 3: Diet and dietary behavior?

7. What do people generally eat in this community (Habitual diet)?
8. Doctors recommend a balanced diet. How do you describe a balanced diet?
9. What is the importance of eating a balanced diet?
10. Are there any challenges in practicing the doctor's advice on a balanced diet?
11. Can you tell us the food items people with diabetes should not eat?
12. Can you describe to me what you understand as a bad diet?

Theme 4: Facilitators of eating a balanced diet

13. Despite the barriers you have mentioned above, how can we encourage people to eat a balanced diet?
14. In terms of culture, what are the community beliefs regarding eating the food items that form a balanced diet?
15. In general, how do people prepared food in this community?

Theme 5: Physical activity

16. What is your understanding of physical activity (any body movement that results in expending energy)?
17. Is regular physical activity important for living a healthy life in the present and future?
18. What are the different physical activities that you take part in from morning to evening?
19. In case you need to meet the recommended level, what do you think could be the acceptable physical activities for the following?
20. In case you need to do more to meet the recommended level, what do you think could stand in your way of meeting and maintaining this goal?
21. What do you think could make it easier for you to meet and maintain the recommended level?
22. When do you think that it is necessary for a person to engage in physical activity?

Theme 6: Risk

23. What are the things that put one at risk of getting diabetes?
24. Who is most at risk of getting diabetes?
25. What can people do at individual level to reduce the risk of getting diabetes?

Theme 7: Risk communication

26. Is raising awareness about diabetes in the communities important?
27. Through what communication channel do you learn about matters of health?
28. Which of the communication channel is most preferable to you at individual and community levels?

Interview/Observation guide for key-informants from the health facility

Theme 1: Preventive services available for general population and high-risk clients

1. Do people who are feeling completely well ever walk in and request for a health check-up?
2. Do you regularly screen people for risk factors for diabetes as a routine procedure in the out-patients clinic or do you do it occasionally for selected clients, or not at all?
3. What services if any does this facility provide to people with risk factors but who do not have diabetes?

Theme 2: Services available for diabetic patients

4. How are diabetes patients mainly detected in this facility?
5. Do you regularly screen some people for early detection of diabetes without them having any complaints related to diabetes?

Theme: Equipment and drugs

6. Are the equipment and drugs available in the facility?
7. What are the types of clients on whom they are most used?

Theme 3: Facility-based and professional care

8. What is the usual number and type of staff in this health facility who provide diabetes care or care for diabetes associated risk factors like hypertension?
9. Please describe the level of geographical access to this health facility by your catchment population Financial access?
10. Accommodation aspects of access for the diabetes clinic: Is opening time, waiting time, and clinic opening days sufficient for the patient load?
11. Is evidence-based guidance for type 2 diabetes care and prevention available for providers? What are the referral pathways from the health facilities to higher levels; how do referrals happen?

Theme 4: Interaction among health actors

12. Are there systems for professionals at different levels to consult each other regarding patient care for diabetes?
13. Do professionals at higher levels train, supervise and exchange evidence-based information with lower level of care regarding diabetes, hypertension and related diseases?
14. What kind of information system exists?
15. Does the system facilitate free exchange of information among actors, integration and a whole person approach?
16. What are the referral pathways for diabetes and hypertension? How do referrals happen between levels?
17. Is there opportunity for multidisciplinary team work within the health facility to discuss problem patients?

Theme 5: Chronic Illness and Person-centered approach

18. Do providers follow a whole person approach?
19. What is the average duration of patient-provider contact in the health facilities per visit?
20. What is the level of awareness / training of providers in patient centered care?

21. Do providers involve patients in decisions about treatment?
22. Do providers promote autonomy among patients?
23. Do providers provide sufficient information to diabetes patients to empower them to take charge of themselves?

Theme 6: Diabetes Self-management

24. To what extent does diabetes self-management happen among people at risk / people with T2DM?
25. Do people have access to diabetes information?
26. Do people have access to medication and self-monitoring tools needed for self-management?
27. How are people being motivated to change their behaviour?
28. Do any particular measures exist to support people in the management of their disease/organisation of their life?

Theme 7: Support services for people with risk factors and diabetes patients

29. What kind of support do they receive from the health facility, friends and family in relation to treatment and implementing diet and physical activity changes?
30. What kind of support do you provide as the facility to patients with diabetes?
31. How do you involve families, friends, fellow patients or community in supporting diabetes patients?
32. What type of community health workers exist in your service zone?
33. Do you have patient groups for chronic diseases in this facility?
34. Do you know of any community-based programs that provide diabetes/chronic care in the Community?

Theme 8: healthcare Utilization

35. What is the utilization rate of formal health services?
36. What is the utilization level for community-based services?
37. Do diabetes related services cover patients' demands?
38. Are patient grievances and complaints being addressed?

Theme 9: Risk communication

39. Do you conduct health education sessions specific to prevention of diabetes among high-risk individuals and community members in general?
40. Do you conduct health education sessions specific management of diabetes to diabetic patients?
41. How are the health education sessions conducted?
42. Please share with us the patient experiences in regard to applying the nutrition/diet and physical activity messages given.
43. What challenges do you experience as health workers in providing health information on prevention and management of diabetes to the community members?

Theme 10: Challenges to providing diabetes care

44. What challenges do you face when providing diabetes related preventive health education and care services for people with diabetes?

Theme 12: Local context, social situation and community

45. Describe the existing initiatives in the community and their role in self-management?

46. Describe who is held accountable for people living with diabetes?
47. To what extend does stigma exist and play a role in the management and care of people living with diabetes?
48. Does the environment in this community allow/enable physical activity?
49. Does the environment in this community allow/enable a healthy diet?

Semi-structures Interview guide for healthcare managers

Theme 1: Facility-based and professional care

1. What is the usual number and type of staff in different levels of health facilities who can provide diabetes care or care for diabetes associated risk factors like hypertension and obesity?
2. Describe in general the level of availability of equipment and infrastructure of facilities in relation to care and prevention of diabetes and its associated risk factors and constraints?
3. Describe in general the level of availability of drugs for care and prevention of diabetes in health facilities and its associated risk factors and constraints?
4. Describe the current level of geographical access to health facilities capable of providing diabetes care in this district?

Theme 2: Interaction among health actors

5. Are there systems for professionals at different levels to consult each other regarding patient care?
6. Do professionals at higher levels train, supervise and exchange evidence-based information with lower level of care?
7. What kind of information system exists?
8. Does the system facilitate free exchange of information among actors, integration and a whole person approach?
9. What are the referral pathways, how do referrals happen between levels?
10. Is there opportunity for multidisciplinary team work within the health facility to discuss problem patients?

Theme 3: Support services for people with risk factors and diabetes patients

11. Do you think people with risk factors in lower level health facilities receive any support in implementing lifestyle measures to control the risk factors?
12. What kind of support do health facilities provide to patients with diabetes?
13. How do health facilities involve families, friends, fellow patients or community in supporting diabetes patients?
14. What type of community health workers exist in this district?

Theme 4: Community support systems for diabetes care and prevention

15. Describe the existing initiatives in the community involved in health promotion?
16. To what extend does stigma exist and play a role in the management and care of people living with diabetes?
17. Does the environment in the communities allow/enable physical activity?
18. Does the environment in the communities allow/enable a healthy diet?

Interview guide for community stakeholders

Theme 1: NCD policy/Program

1. Describe the main strategic lines and choices of the NCD policy and specifically of the Diabetes control policy/programme

Theme 2: Services related to diabetes prevention and care in health facilities

2. How would you describe the current availability of services for prevention diagnosis and management of type 2 diabetes in Uganda at different levels?

Theme 3: Challenges and gaps in NCD prevention and care

3. What do you think are the current constraints/set-backs/challenges in the NCD policy in terms of clarifying what needs to be done for NCD prevention in the country?
4. What are the gaps in provision of NCD care in Uganda with regard to the following?

Interview guide for policy makers at the Ministry of Health

Theme 1: Policy Framework

5. Could you please describe the Policy framework for diabetes care and prevention in the country?
6. Could you comment on the readiness of Uganda as a country to address the prevention and management of NCD's

Theme 2: NCD policy

7. Describe the main strategic lines and choices of the NCD policy and specifically of the Diabetes control policy/programme
8. How did this policy/programme come about?
9. How are the partnerships with NGOs and private providers?
10. Are influential political leaders being identified and involved in health prevention and promotion?
11. Is there a consistent and sustainable financing policy integrated across traditionally disparate disease categories and levels of care?

Theme 3: Programmes

12. Which priority NCDs are targeted in Uganda?
13. How is Uganda addressing the problem of NCD's at various levels Community?

Theme 3: Policies and programs related to NCDs but indirectly

14. Please describe policies/programs/guidelines to regulate or guide the food, alcohol, Tobacco and physical activity?

Interview/FGD guides: South Africa

Interview/FGD guide for people with type 2 diabetes

Theme 1: Health seeking behaviour

1. Please tell us when did you last feel unwell?
2. What symptoms did you have when you were unwell?
3. Where do you go when you feel unwell?
4. How often do you visit a health facility for a routine check-up even when you are feeling well?
5. How do you perceive diabetes?
6. What mode of treatment is given for diabetes?
7. How do you manage /control non-communicable diseases like diabetes and hypertension?
8. What would you like to do to improve care for people with diabetes in your community? In the country?
9. What can be done to prevent or control diabetes in your community?

Theme 2: Lifestyle behaviour (diet / physical activity)

1. From what you have been told, what do you know about the cause/s, prevention and management of diabetes?
2. What do you think about your own chances of getting diabetes?
3. How do you think that eating can contribute to diabetes?
4. How would you describe a healthy diet?
5. How do you think the people in your community eat healthily?
6. How do you think the environment where you live makes it possible to eat healthily?
7. How important is it to engage in physical activity?
8. From your knowledge, what are some of the benefits of regularly engaging in physical activity?
9. In general, what are the things that prevent people from exercising on a regular basis?
10. Would you like to be more physically active, and if so, what would you need to become more physically active?

Theme 3: Health education needs

1. What information did you receive about diabetes?
2. What do you think about the information you received?
3. What is your opinion about raising awareness about diabetes in the communities?
4. What kind of information about diabetes would be most needed?
5. What other support besides information would you need in order to make changes in your lifestyle?
6. How should communication on, and other support for prevention and control of diabetes be packaged and passed on to an individual and the community?

Interview/FGD guide for people at risk

Theme 1: Health seeking behaviour

1. Please tell us when did you last feel unwell?
2. Have you ever sought health care?

1. What do you know about non-communicable disease?
2. What have you heard people in your community say about diabetes?
3. How do you manage diabetes in your community?
4. Where do people suffering from diabetes seek care?

Theme 2: Lifestyle behaviour

1. What do people in your community think about being overweight?
2. What things or activities can people do to avoid overweight / diabetes?
3. What foods can contribute to diabetes and if so, what kind of food increases the risk of diabetes?
4. How would you describe a healthy diet?
5. How do you think that people in your community eat healthily?
6. How do you think that the environment where you live makes it possible to eat healthily?
7. How important do you think is engaging in physical activity?
8. From your knowledge, what are some of the benefits of regularly engaging in physical activity?
9. In general, what are the things that prevent people from exercising on a regular basis?
10. How do you perceive your physical activity today?
11. What do you think you need to become more physically active?

Interview/ FGD guide for community members

Theme 1: Health seeking behaviour

3. Please tell us when did you last feel unwell?
4. Have you ever sought health care?
5. What do you know about non-communicable disease?
6. What have you heard people in your community say about diabetes?
7. How do you manage diabetes in your community?
8. Where do people suffering from diabetes seek care?

Theme 2: Lifestyle behaviour (diet / physical activity)

9. What do people in your community think about being overweight?
10. What things or activities can people do to avoid overweight / diabetes?
11. What foods can contribute to diabetes and if so, what kind of food increases the risk of diabetes?
12. How would you describe a healthy diet?
13. How do you think that people in your community eat healthily?
14. How do you think that the environment where you live makes it possible to eat healthily?
15. How important do you think is engaging in physical activity?
16. From your knowledge, what are some of the benefits of regularly engaging in physical activity?
17. In general, what are the things that prevent people from exercising on a regular basis?
18. How do you perceive your physical activity today?
19. What do you think you need to become more physically active?

Interview/FGD guide for health care providers

Theme 1: Introductory questions

1. What is your role and how long have you worked in this role?
2. You earlier mentioned that you were a Health manager / Doctor / Nurse / Nutritionist? What is a typical day for you at work? What activities do you do? Who do you work with?

Theme 2: Existing health services for diabetic and pre-diabetic persons

3. Please explain how you identify these patients and enroll them for treatment.
4. What kind of treatment services do you offer these patients?
5. What about the pre-diabetic individuals, how do you identify them?
6. How do these diabetic patients actually access the services they are entitled?
7. How is the access for the pre-diabetic individuals, it is the same as diabetic patients? Elaborate please

Theme 3: The diabetes patients

8. Please describe the typical diabetes patients attending health services here
9. Who are the patients that face more difficulty in accessing these services?
10. They say that diabetes is not curable and that in order to live longer and healthy the patient must take responsibility for his behavior and wellbeing in regard to lifestyle. How do you communicate this to your patients? What are some of the messages, tools, materials and strategies you use?
11. What responses do you get from your patients regarding these messages, do they comply? What are their concerns?
12. What challenges do you encounter when delivering these messages?

Theme 4: Healthcare provider experiences, challenges and facilitators

13. What are the challenges you encounter treating diabetes clients as opposed to other patients with lifestyle related illnesses? What is similar? And what is different?
14. How do you cope with these barriers? Please elaborate

Theme 5: Recommendations

15. What strategies, messages, tools, etc., would you recommend be sustained and/or scaled up? Please provide a justification for your response.
16. What strategies, interventions, tools should be discontinued? Why?

Theme 6: Diabetes guidelines

17. Do you follow any guidelines when managing both diabetic and pre-diabetic patients? Please describe these guidelines
18. What has been your experience using the diabetes guidelines? Are they easy to use? Please explain
19. Are there any issues that we might not have discussed that you would like to add?

Interview guide for community key informants

Theme 1: Understanding community structures

1. The term 'community' can mean different things to different people, so what do you understand by the word community?
2. Why do you think do people spend time in communities in your setting?

Theme 2: Experience and motivation for community engagement

3. Please tell us about your social circle?
4. How are activities coordinated within the community?
5. How important is language in your networks?

Theme 4: Community support

6. Communities/networks can sometimes act as a source of support to members; do you feel that is the case here?
7. What would stop people from seeking support?
8. Do you think that the support a network provides to someone who is sick is different from other townships?
9. Where does the network look for support if / when needed?

Theme 5: Community influence on behavior

10. When it comes to behavior, do you think the community / network influences healthy behavior?
11. What activities are available to promote healthy behavior in your community?
12. Can you think of an example where behaviors or decisions changed because of what people in the community were doing?
13. How does the community discuss conditions like diabetes / heart disease / high blood pressure?
14. Which information is shared? What is considered too private to share? Are there any differences in gender?

Theme 6: Final questions

15. What are the two-three potential changes in the network that could strengthen support for members with for example diabetes or high blood pressure?

16. What are two / three constraints in achieving this change?

Interview guide for community leaders who are active in a network

Theme 1: Relationship with the community

1. How long have you worked with these communities?

Theme 2: Community group structure

1. Which groups / networks /associations do you know of?
2. How did these groups come about?
3. Are these groups a good reflection of their community? How do people hear about these groups?
4. How are group leaders selected?
5. What are the main reasons for members to be active?
6. What language is used in the group?

Theme 3: Community support

7. What kind of support do these groups provide to individual members?
8. Who decides on the type of support that is provided or how is this decided?

Theme 4: Community influence on behavior

9. Have you witnessed occasions whereby people joined a group and their behavior changed?
10. Can you think of a group that has gradually incorporated activities into their habits?
11. Are there other networks who work in a similar way in the community we could be put in touch with?

Theme 5: Final questions

12. What are the two-three potential changes in the network that could strengthen support to members with for example diabetes or high blood pressure?
13. What are the two-three constraints in achieving this change?

Interview/FGD guides: Sweden

Semi-structured interview guide for people with type 2 diabetes

Theme 1: Perceptions of type 2 diabetes diagnosis

1. Please describe the period just before you got the T2D diagnosis: how did you feel?
2. Tell us about the consultation when you received your diagnosis?
3. After you had been diagnosed, how did you react?
4. If you were to compare the reactions you got when you received diabetes diagnosis do you think that they would be the same as if you got a diagnosis of another chronic disease?
5. If you were to compare the reactions you got when you received the diabetes diagnosis with the reactions you have noticed that other people with diabetes got, do you think that these reactions are similar? What about people with high blood pressure – what reactions do they get? What about people with other chronic diseases?

Theme 2: Diabetes care, patient and provider interactions

6. Can you describe your contact with the healthcare center?
7. During your visits to the health center what do you talk about?
8. Please describe the information you receive during consultations at the healthcare center
9. What changes have you made in relation to your diet, smoking, physical activity after your diagnosis?

Theme 3: Experiences of diabetes self-management

10. Can you describe what you do on a daily basis to manage your condition?
11. Please describe how you feel and what you do to keep your blood sugar stable on a good day?

Theme 4: Support for self-management

12. If you think about your everyday life with diabetes – describe the support you have received?
13. What challenges have you faced during the management of your condition?

Semi-structured interview guide for people with persons at risk of diabetes (Prediabetes)

Theme 1: Perceptions of risk for diabetes

1. Please describe the period just before you got to know that you were at risk of diabetes
2. Tell us about the consultation when you received your diagnosis?
3. After you had been diagnosed, how did you react?
4. If you were to compare the reactions you got when you received prediabetes diagnosis do you think that they would be the same as if you got a diagnosis of diabetes or another chronic disease?
5. If you were to compare the reactions you got when you received the diagnosis with the reactions you have noticed that people with diabetes got, do you think that these reactions are similar? What about people with high blood pressure – what reactions do they get? What about people with other chronic diseases?

Theme 2: Care for persons at risk of diabetes and interaction with healthcare providers

6. Can you describe your contact with the healthcare center?
7. During your visits to the health center what do you talk about?
8. Please describe the information you receive during consultations at the healthcare center

9. What changes have you made in relation to your diet and physical activity after your diagnosis?

Theme 3: Experiences of coping with the risk of diabetes

10. Can you describe what you do on a daily basis to manage your condition?

11. Please describe how you feel and what you do to keep your blood sugar stable on a good day?

Theme 4: Support for persons at risk of diabetes

12. If you think about your everyday life as being at risk of T2D– describe the support you have received?
13. What challenges have you faced during the management of your condition?

Semi-structured interview guide for healthcare professionals and health managers

Theme 1: Diabetes care, patient and provider interactions

1. How is diabetes care organized at this healthcare center?

2. Describe the typical patient seeking diabetes care here?

3. How do patients react when diagnosed with T2D?

4. Please describe the type of treatment programs you offer newly diagnosed patients?
5. How do patients react to becoming diagnosed with diabetes?

6. How do you describe self- management to the patients? How do they understand it?

Theme 2: Caregiver's experiences and support for self-management

1. Do you offer other types of support to the T2D patients?
2. What challenges do you encounter in the treatment of patients with diabetes as compared to patients with other lifestyle-related diseases?

3. How do you view the need for diabetes prevention and primary health care treatment in areas with a large proportion of migrants in relation to areas where most people are born in Sweden?

4. Perceptions of disease causes can vary between different cultures and it can be difficult for the doctor and the patient to understand each other- Do you think this is a problem in diabetes care here?

5. To what extent do you think that proper care and support are offered to diabetes patients?
6. If a patient has trouble following treatment and recommendations, how is that managed?

Semi-structured interview guide for local network leaders/community members

Theme 1: Perceptions of community

1. Community can mean different things for different cultures - How do you understand the term community?
2. What networks are found within this community?
3. How do people become members in these networks?
4. How are activities coordinated?
5. Language is seen to be an important means to communicate – In what way does language affect people's participation in the proposed networking activities in your area?

Theme 2: Perceptions of health and care

6. What does it mean for you to "feel healthy"?
7. What is your experience seeking care at the primary health care center?
8. If you think about the people you know - how do they choose which healthcare center they use?
9. What would you like to see changed in health care?

Theme 4: Support for persons with diabetes within the community

10. Have you heard of type 2 diabetes before this study?
11. What types of food do people in your network usually eat?
12. What are the common physical activities most people do in your local area?
13. What do you think people in the community are doing to prevent diabetes?
14. In terms of diabetes prevention, what is the role of the network?
15. What do you suggest that society in general can do to improve the community's eating habits and physical activity?
16. If you had a diabetic patient today, how would you support them to ensure that they follow their treatment plan from the healthcare center?
17. How do you expect the rest of society to support you in taking care of this patient?

Semi - structured interview guide with health actors at national and regional institutions

Introductory questions

1. What is the mission your organization?
2. What is your role in the organization?

Theme 1: Experiences of interacting with the local community

3. What activities is your organization engaged in with in the community?
4. How does your organization interact with community networks?

Theme 2: Awareness of diabetes burden in the community

5. What have you heard being said about diabetes in the community?
6. How do people with diabetes in the community manage the disease?

Theme 3: Health promotion and diabetes prevention

7. How is your organization working round the communities to promote health?
8. What programs is your organization involved in to prevent diabetes in the community?
9. How can your organization support community members with prediabetes and type 2 diabetes to make lifestyle changes?
10. Cooperation between various organizations in society- How do you think you to cooperation can be established between organizations for to support to lifestyle changes so as to prevent diabetes?
11. What challenges do you face in your work with the community?
12. What strategies would you recommend for local and regional authorities to interact and engage with communities to prevent diabetes
13. Which Other actors do you think would be relevant in development of these strategies.
