# Supplementary material for: Diabetes self-management in three different income settings: Cross-learning of barriers and opportunities
Source: PLoS One. 2019 Mar 19;14(3):e0213530. doi: 10.1371/journal.pone.0213530 (PMC6424475; doi:10.1371/journal.pone.0213530)
Supplement: S2 Appendix — (DOCX) [file pone.0213530.s002.docx]

#### Supplementary file 2:

#### Topic Guide for situation analysis

#### Cross-lessons on the implementation of T2D Self-Management

####

## Table of contents

Table of contents 2

A. Individuals with (pre) T2DM and their families 3

B. Local health system and community actors related to T2DM 3

C. Optimal T2DM services 5

D. Utilization of health care and community-based services, self-management practices 8

E. Output indicators of T2DM management: Control of risk factors, glycaemic control, patient satisfaction, wellbeing, etc 10

F. Local context (field site/district/town area), social situation and community 10

G. Distal context (country profile) 11

## Individuals with (pre) T2DM and their families

(Results can be presented separately for people with pre-diabetes and for people with diabetes)

Perceptions of wellbeing & disease, attitudes towards prevention activities like lifestyle modification, health seeking & care practices, gender differences in notions of wellbeing, etc.

A synthesis of the findings from the focus groups/in-depth interviews can be provided here, complemented with information from other sources (grey literature, experts’ knowledge).

A description of relevant social determinants of the target population can be provided here.

## Local health system and community actors related to T2DM

### Facility-based and professional care

This concerns the first line health center, the health post, outreach teams, first-level referral hospitals…

| **Topics** | **Source of information** | **Response** |
| --- | --- | --- |
| Type of care provided per level and type (public, private, for profit/non-for profit – if applicable) relevant for T2DM | Routine health information system, expert knowledge |  |
| Number and type (training, status) of staff | Routine health information system |  |
| Equipment and infrastructure of facility | Observation, Routine health information system, provider interviews |  |
| Geographical access (roads and barriers, average distance (if available), available transportation, etc.) | Patient interviews, grey literature, expert knowledge |  |
| Financial access (OOP (formal, informal), transportation fee, indirect costs) | Patient interviews, grey literature, expert knowledge |  |
| Are drugs and monitoring devices related to T2DM available and affordable? | Patient interviews, grey literature, expert knowledge |  |
| Accommodation aspects of access: opening time, home visits, waiting time, patient perceptions, language | Patient interviews, grey literature, expert knowledge |  |
| Acceptability aspects of access: religion, perceived quality | Patient interviews, grey literature, expert knowledge |  |
| Is evidence-based guidance available for providers? (guidelines, flow charts, training) | Country guidelines, expert knowledge |  |

### Community providers relevant for T2DM

*Community providers in the local health system: Describe what kind of formal and informal community providers and community groups exist that are or can be relevant for T2DM management and prevention.*

| **Topics** | **Source of information** | **Response** |
| --- | --- | --- |
| Type of care provided by each type of community-based provider | Expert opinion, policy documents, patient interviews, community interviews |  |
| How are these community groups related to the community: proximity, status … | Expert opinion, policy documents, patient interviews, community interviews |  |
| How are these groups related the local health system? | Expert opinion, policy documents, community interviews |  |
| Do these groups receive support from the (local) government? Describe | Expert opinion, policy documents, community interviews |  |

### Interaction among health actors (in particular community health actors and professional providers)

| **Topics** | **Source of information** | **Response** |
| --- | --- | --- |
| Describe the communication/coordination between and within both groups (community and professional providers) | Provider interviews, community interviews |  |
| Do professionals train, supervise and exchange evidence-based information with community health actors? Explain | Provider interviews, community interviews |  |
| What kind of information system does exist? (Patient cards, medical files, digital information?) Does the system facilitate free exchange of information among actors, integration over time and a whole person approach? | Provider interviews, health system documents |  |
| What are the referral pathways, how do referrals happen (e.g. communication)? | Provider interviews, health system documents, community interviews, patient interviews |  |
| Is there opportunity for multidisciplinary team work within the health facility to discuss problem patients? | Provider interviews |  |

## Optimal T2DM services

### Minimal package of care for T2DM

*Describe if the care components mentioned in this table are provided and at which level, you can also add more details if necessary.*

| **IDF care package recommendations (for Limited Care settings)** | **Specific task** | **Currently done? Y/N/partially** | **At which level/platform (community, facility, self-management)** | **Details** |
| --- | --- | --- | --- | --- |
| **Case detection:**  Opportunistic screening  and limited to high-risk individuals in very limited  settings | Passive case detection |  |  |  |
|  | Active screening of high risk individuals |  |  |  |
|  | Enhanced case-finding |  |  |  |
| **Diagnosis:** fasting blood glucose (plasma / capillary) | PPPG or FPG |  |  |  |
| **Glucose monitoring** | Blood test HbA1C |  |  |  |
|  | Blood test glycaemia |  |  |  |
|  | capp. test glycaemia |  |  |  |
| **Self-monitoring** for people on insulin | Capp. test glycaemia |  |  |  |
| **Oral anti-diabetics** | Start-up and change |  |  |  |
|  | Provide medication |  |  |  |
|  | Compliance monitoring |  |  |  |
| **Insulin** | Start-up and adaptation |  |  |  |
| **Blood pressure control:** at least 1x py, < 130/80 | Check  adaptation treatment & regimen |  |  |  |
| **Cardiovascular risk-assessment** | Blood test (lipids) |  |  |  |
|  | Start-up statin |  |  |  |
| **Eye screening** | Fundoscopy and visual acuity |  |  |  |
| **Kidney damage** | Blood and urine test |  |  |  |
|  | Start-up ACE |  |  |  |
| **Foot care** | Annual check with mono-filament |  |  |  |
| **Nerve care** | Opportunistic screening? |  |  |  |
| **Life-style management support** |  |  |  |  |
| **Psycho-social support** |  |  |  |  |
| **Treatment of complications** | Glycaemia deregulation (hypo, hyper)  Micro or macro vascular complica-tions  (e.g. diabetic ulcer) |  |  |  |

### Chronic Illness and Person-centered approach

| **Topics** | **Source of information** | **Response** |
| --- | --- | --- |
| Providers follow a whole person approach (e.g. take into account the psychosocial context) | Observation of patient-provider interaction |  |
| Average duration of patient-provider contact | Observation of patient-provider interaction |  |
| Awareness / training of providers in patient centered care | Provider interview  Patient interview |  |
| Do providers involve patient in decisions about treatment? Do they provide time for this? | Expert knowledge, patient interviews, provider interviews |  |
| Do providers promote autonomy among patients (empower patients to manage their disease and make their own decisions)? | Expert knowledge, patient interviews, provider interviews |  |

## Utilization of health care and community-based services, self-management practices

### Diabetes Self-management (site-specific)

| **Topics** | **Source of information** | **Response** |
| --- | --- | --- |
| To what extent does diabetes self-management happen among people at risk / people with T2DM? Adaptation of diet, physical activity, follow-up of blood pressure, medication compliance, glucose monitoring, foot care, visiting providers, knowledge what to do in emergencies (hypoglycaemia). | Expert knowledge, patient interviews, provider interviews, literature |  |
| Do people have access to information: explanation of diabetes, possible prevention measures, the expected course, expected complications, and effective strategies to prevent complications and manage symptoms? How is this information provided and by whom? | Expert knowledge, patient interviews, provider interviews, literature |  |
| Do people have access to medication and self-monitoring tools needed for self-management? | Expert knowledge, patient interviews, provider interviews, literature |  |
| How are people being motivated to change their behaviour? By whom? Do they use a particular strategy? | Expert knowledge, provider interviews |  |
| Do any particular measures exist to support people in the management of their disease/organisation of their life? (e.g. social benefits, wheelchair, adapted work) | Expert knowledge, patient interviews, provider interviews, literature |  |

### Utilization

| **Topics** | **Source** | **Response** |
| --- | --- | --- |
| Utilization rate of formal health services (if possible related to diabetes care) | Health system data |  |
| Utilization rate of Community-based services (if possible related to diabetes care) | Community interviews/data |  |
| Do services cover patients’ demands? (E.g. hours open, waiting times, perceived quality, …) | Patient interviews, provider interviews |  |
| Are the necessary drugs available at the health centre/post? | Patient interviews, provider interviews |  |
| Are patient grievances and complaints being addressed, do avenues exist to address these? | Patient interviews, provider interviews |  |

## Output indicators of T2DM management: Control of risk factors, glycaemic control, patient satisfaction, wellbeing, etc

| glycaemic control | Proportion of diabetics on oral anti-diabetics |  |
| --- | --- | --- |
|  | Average Hb1AC of diagnosed diabetics |  |
| Risk factors | Proportion of patients with a normal blood pressure |  |
|  | Proportion of patients following a diet |  |
|  | Proportion of patients doing physical activity |  |

## Local context (field site/district/town area), social situation and community

### Existing community networks, social anchors

Describe their existence and potential relevant to diabetes care and management

### Expected support from family, relatives

To what extend can people living with diabetes expect support from their family, are they still accepted in their families, does status in the family play a role in this?

### Community promotion of healthy habits (diet, physical activity)

Describe the existing initiatives.

### Community position towards self-management of health and care

Is this accepted, promoted, enabled?

### Community/cultural position towards accountability

Describe who is held accountable for people living with diabetes, the patient her/himself, the family, the community, the health care system, or god?

### Community/cultural position towards T2DM (stigma)

To what extend does stigma exist and play a role (could be positive as well as negative) in the management and care of people living with diabetes.

### Environmental elements

Is the study setting rural or urban?

Does the environment allow/enable physical activity?

Does the environment allow/enable a healthy diet?

## Distal context (country profile)

### Health system

##### General characteristics of the health system

Describe the overall structure of the health system (main actors, structure and tiers, relations and interactions among main actors and between the different levels, incl. referral system, the provision, degree of integration, … )

Governance structure: describe the national / local governance structure (+ degree of decentralization), relationship between public and private providers (accreditation, regulation, cooperation, …)

Health financing: Describe health financing (public-private expenditure, OOP, pooling mechanisms – health insurance)

##### Health policy

- Describe recent developments in national health policies
- Describe recent health sector reforms
- To what extent is the policy environment transparent, are citizens/communities involved in policy formulation (participation).
- NCD policy:
  - Describe the main strategic lines and choices of the NCD policy and specifically of the Diabetes control policy/programme
  - How did this policy/programme come about?
  - Who are the relevant stakeholders?
  - Are cross-sectorial and multilevel partnerships being promoted?
  - How are the partnerships with NGOs and private providers?
  - Are influential political leaders being identified and involved in health prevention and promotion?
  - Is there a consistent and sustainable financing policy integrated across traditionally disparate disease categories and levels of care?

### NCD country indicators (see WHO country profiles NCD)

| **Indicator** |  |
| --- | --- |
| Has an operational multisectoral national policy, strategy or action plan that integrates several NCDs and shared risk factors |  |
| Has an operational NCD unit/branch or department within the Ministry of Health, or equivalent |  |
| Has an operational policy, strategy or action plan to reduce T2DM incidence |  |
| Has an operational policy, strategy or action plan to promote physical activity |  |
| Has an operational policy, strategy or action plan to promote healthy diets |  |
| Has evidence-based national guidelines/protocols/standards for the management of major NCDs through a primary care approach |  |
| Has an NCD surveillance and monitoring system in place to enable reporting against the nine global NCD targets |  |
| Proportion of deaths NCDs are estimated to account for |  |
| The probability of dying between ages 30 and 70 years from the 4 main NCDs |  |
| Current tobacco smoking |  |
| Total alcohol per capita consumption, in litres of pure alcohol |  |
| Raised blood pressure |  |
| Obesity |  |
| Are doctors and nurses being trained in T2DM? |  |
| Does the essential package of care includes T2DM drugs and other related costs? |  |

### Demographic information of the study site

| **Indicator** |  |
| --- | --- |
| **General** |  |
| Total population |  |
| Population density |  |
| Age distribution |  |
| Poverty rate |  |
| Rate of unemployment |  |
| Main occupations |  |
| Cultural characteristics |  |
| Religion |  |
| Minority groups |  |
| Mobility |  |
| **T2DM related** |  |
| Reported incidence of T2DM |  |
| Estimated incidence of T2DM |  |
| Reported incidence of pre-diabetes |  |
| Reported incidence of pre-diabetes |  |
| Obesity rate |  |
| Diet |  |
| Physical activity patterns |  |

### National indicators

| **Indicator** |  |
| --- | --- |
| GDP per capita in PPP |  |
| Economic growth |  |
| Total health expenditure THE |  |
| Per cent of THE government contribution for health |  |
| Per cent of THE Out-of-pocket |  |
| Country income group (low-middle-high) |  |
| Life expectancy at birth (in years) |  |
| Infant Mortality Rate (IMR) per 1,000 live births |  |
